# Coronary Microvascular Disease Early After Myocardial Infarction: Diagnostic Approach and Prognostic Value—A Narrative Review

**DOI:** 10.3390/biomedicines13061289

**Published:** 2025-05-23

**Authors:** Stefanos Sokratous, Andreas Mitsis, Elina Khattab, Dimitrios Karelas, Nikolaos Velidakis, Nikolaos P. E. Kadoglou

**Affiliations:** 1 Medical School, University of Cyprus, 2029 Nicosia, Cyprus; 2 Department of Cardiology, General Hospital of Nicosia, 2031 Strovolos, Cyprus; 3 2nd Cardiology Department, “Korgialenio–Benakio” Red Cross Hospital, 11526 Athens, Greece

**Keywords:** coronary microvascular disease (CMVD), acute myocardial infarction (AMI), microvascular obstruction (MVO), index of microcirculatory resistance (IMR), percutaneous coronary intervention (PCI), coronary flow reserve (CFR), major adverse cardiovascular events (MACE)

## Abstract

Coronary microvascular disease (CMVD) is not an uncommon complication after acute myocardial infarction (AMI), independent of prompt revascularization. It is a serious yet underdiagnosed disease that has a major impact on patient outcomes. Even when the infarct-related artery is successfully revascularized, a significant percentage of patients still have compromised microvascular circulation, which is linked to higher cardiovascular mortality and hospitalization for heart failure. The well-known invasive methods, such as the index of microvascular resistance (IMR) and the coronary flow reserve (CFR), have been considered as gold standards. However, they are constrained by their hazards and complexity. Non-invasive techniques, such as echocardiography Doppler for CFR assessment, positron emission tomography (PET), cardiac magnetic resonance imaging (CMR), and some other techniques provide alternatives, but their accessibility, cost and implementation during the peri-AMI period raise obstacles to their wider use. This review highlights both invasive and non-invasive modalities as it examines the diagnostic methods and prognostic significance of CMVD development early after AMI. Enhancing long-term results in this high-risk population requires a thorough understanding of pathophysiology and a commitment to larger diagnostic and prognostic studies for CMVD.

## 1. Introduction

The coronary vasculature is usually divided into macrovascular and microvascular circulation. The latter involves vessels smaller than 0.5 mm in diameter, which constitute 90–95% of the whole coronary artery network, but are not usually seen in both invasive and non-invasive coronary angiography [1]. Coronary microvascular disease (CMVD) is a crucial yet often underdiagnosed complication following acute myocardial infarction (AMI), encompassing both ST-segment elevation myocardial infarction (STEMI) and non-ST elevation myocardial infarction (NSTEMI). The diagnosis of CMVD is of paramount clinical importance, and the available diagnostic modalities, either invasive or non-invasive, have shown high variability in their results. The thermodilution-based index of microvascular resistance (IMR) is regarded as the gold standard among invasive methods, while additional wire-based techniques such as coronary flow reserve (CFR) and fractional flow reserve (FFR) have also been explored [1]. These invasive approaches carry procedural risks and are constrained by time-sensitive applicability. Non-invasive alternatives, including echocardiography-based CFR, cardiac positron emission tomography (PET), and cardiac magnetic resonance (CMR) imaging, offer valuable insights into microvascular function but face limitations regarding variable accuracy, low accessibility, high cost, and lack of standardization [2].

CMVD is linked to a higher risk of major adverse cardiovascular events (MACE), including mortality, hospitalization for heart failure, and/or acute coronary syndromes in the general population [3]. In the case of patients with AMI, a growing number of studies have demonstrated the negative impact of CMVD presence on AMI prognosis despite an adequate restoration of epicardial coronary blood flow after primary percutaneous coronary intervention (PPCI) [4]. Even in the case of revascularized and fully-patent coronary arteries, CMVD can cause persistent myocardial damage and electrical instability, which are of clinical relevance [5]. A meta-analysis of retrospective studies demonstrated that severe CMVD, assessed during PCI in STEMI patients, was significantly associated with higher incidence of MACEs [6]. Hence, CMVD can serve as an early index of poor prognosis in AMI patients, outlining the need for better diagnostic tools and targeted therapies [2,7]. Unfortunately, the diagnostic investigation of CMVD has not been included in routine clinical practice and mostly remains a research objective in selected centers [8]. All these diagnostic obstacles undermine the clinical significance of CMVD in AMI patients and future studies are required to reach a firm conclusion.

Given these implications, this review aims to evaluate the available diagnostic modalities for CMVD, critically appraise their clinical applicability, and analyze their prognostic significance to develop a new strategy for post-AMI CMVD assessment.

## 2. Search Methods

We searched MEDLINE (via Ovid SP), EMBASE (via Ovid SP), and the Cochrane Library from 1990 to December 2024, using a predefined search strategy. To identify any additional published and ongoing studies, we searched the Science Citation Index and checked the references of original research studies, previous systematic reviews or meta-analyses, and literature reviews with related subjects. The following search terms, in titles and abstracts, including Medical Subject Headings (MeSH), were used: coronary microvascular disease, coronary microvascular dysfunction, coronary flow reserve (CFR), coronary flow velocity reserve (CFVR), acute myocardial infarction (AMI), ST-elevation myocardial infarction (STEMI), non ST-elevation myocardial infarction (NSTEMI), acute coronary syndrome (ACS), myocardial infarction with non-obstructed coronary arteries (MINOCA), major adverse cardiac events (MACEs), death, cardiovascular death, cardiac death, myocardial infarction, hospital admission, and/or coronary revascularization.

## 3. Potential Mechanisms of CMVD After AMI

Post-AMI CMVD represents the failure to restore adequate blood flow within the coronary microcirculation in regions previously affected by ischemia, even after successful reperfusion of the epicardial arteries. CMVD plays a vital role in maintaining myocardial perfusion [9]. Mechanistically, CMVD arises from a complex interplay of ischemic damage per se, ischemia-reperfusion injury, endothelial dysfunction, inflammatory cascades, and distal embolization in the context of patient-specific risk factors [10]. Each of these mechanisms uniquely contributes to microvascular dysfunction or even microvascular obstruction (MVO), characterized by the inability of blood to adequately perfuse specific regions of the myocardium, even with patent epicardial coronary arteries. The clinical impact of CMVD becomes evident in post-AMI patients, where an impaired microvascular function exacerbates ischemic myocardial damage [11].

### 3.1. Ischemia and Endothelial Dysfunction

Ischemia-induced microvascular injury serves as the foundation. Prolonged ischemia during AMI initiates a cascade of cellular and extracellular disturbances that culminate in cardiomyocyte necrosis and apoptosis [12]. The depletion of ATP impairs ion pump function and leads to intracellular calcium overload, acidosis, and reactive oxygen species (ROS) production [13]. Presumably, these changes result in myocardial cell swelling and interstitial oedema, mechanically compressing the microvascular network [14]. Additionally, the accumulation of metabolic byproducts, such as lactate and degraded nucleotide phosphates, creates an osmotic gradient further exacerbating cellular swelling and microvascular blood flow obstruction [15]. In parallel, reperfusion per se precipitates microvascular injury. Restoration of myocardial oxygen supply leads to a surge in ROS, which compromises cellular viability [16]. Furthermore, ROS trigger pathways such as ferroptosis and necroptosis, which release damage-associated molecular patterns (DAMPs) and amplify inflammation [17]. This self-propagating cycle of oxidative stress and inflammation worsens MVO.

The endothelium, which plays a pivotal role in regulating vascular tone and hemostasis, undergoes significant functional impairment post-AMI [18]. It is affected by reduced nitric oxide (NO) bioavailability, heightened oxidative stress, and upregulated inflammation. Endothelial cell damage during reperfusion induces intramyocardial hemorrhage (IMH) and interstitial oedema, further impairing myocardial perfusion [19]. The infiltration of neutrophils and platelet–leukocyte aggregation stimulate the release of vasoconstrictors [16]. Together with microvascular injury, a persistent endothelial dysfunction increases infarct size, hinders myocardial recovery, and promotes adverse ventricular remodeling [20].

### 3.2. Inflammation

Inflammation represents a pathogenic response in CMVD. Following myocardial injury, released cytokines such as interleukin-6 (IL-6) and tumor necrosis factor-alpha (TNF-α) recruit immune cells to clear necrotic debris [21,22]. Moreover, the release of DAMPs during regulated cell death, such as necroptosis and pyroptosis, amplifies systemic inflammation [7]. As a result, excessive or prolonged inflammation damages the microvasculature through endothelial apoptosis, increased permeability, and the deposition of extracellular matrix proteins, leading to the aforementioned adverse effects on microvascular dysfunction. In turn, the development of fibrosis reduces capillary density and impairs myocardial perfusion.

### 3.3. Percutaneous Coronary Intervention and Distal Embolization

PCI itself can contribute to CMVD [2,23]. While restoring blood flow in the infarct-related epicardial coronary artery, manipulation during PCI, such as crossing wires, balloon pumping, and stent implantation, can induce myocardial injury and the no-reflow phenomenon [13]. Mechanisms such as distal embolization, endothelial blebbing, myocardial swelling, and IMH, alongside the formation of platelet–leukocyte plugs, further contribute to coronary MVO [9]. Among these, distal embolization during PCI poses a central role in CMVD [24]. The mechanical disruption of atherosclerotic plaques can release thrombotic and plaque debris into the coronary microcirculation, causing occlusion at the capillary level [25]. These microemboli not only block perfusion but also trigger localized inflammation and microinfarction [26]. The extent of myocardial damage correlates with the size of the emboli and the degree of MVO [19]. Furthermore, inflammatory processes in the adjacent viable myocardium impair contractility and propagate CMVD, contributing to adverse outcomes such as arrhythmogenesis [20].

### 3.4. Patient-Specific Factors

**Figure 1:**
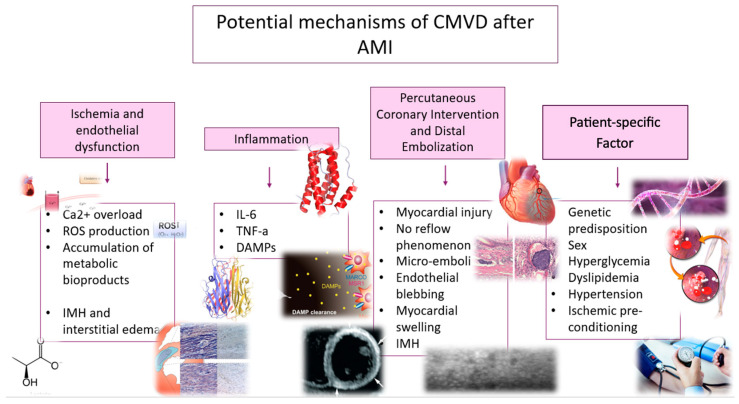
Potential mechanisms of coronary microvascular disease after acute myocardial infarction.

Individual susceptibility and preexisting risk factors further influence the development of CMVD. Genetic predispositions significantly influence microvascular response, with polymorphisms in genes like *VEGFA* and *CDKN2B-AS1* linked to an increased risk of MVO [27]. Additionally, sex-specific allelic variants in *MYH15*, *NT5E*, and *VEGFA* have been shown to differentially affect CMVD risk in men [28]. Hyperglycemia worsens MVO risk by promoting leukocyte plugging and platelet hyperactivity, impairing endothelial vasodilation, and exacerbating microvascular plugging [29]. Dyslipidemia further impairs endothelial function, reduces NO bioavailability, and increases oxidative stress [30]. Hypertension may deteriorate endothelial function, coronary vasodilation, and structural remodeling of microvessels [22]. Additionally, advanced age independently predisposes individuals to CMVD, reflecting cumulative declines in vascular function [20]. Finally, ischemic preconditioning, notably through pre-infarction angina, provides a protective mechanism by reducing cardiomyocyte death and MVO via adaptive responses to ischemic stress. However, its benefits may be diminished by preexisting risk factors [31]. All potential mechanisms are depicted in Figure 1.

## 4. Diagnostic Methods

Several invasive and non-invasive methods have been proposed for CMVD diagnosis early after AMI. The former methods are further subdivided into angiography-based techniques, pressure-wire-based methods (either with thermodilution or Doppler techniques), and novel angiography-derived markers using computational flow dynamics using different equipment [32]. Table 1 highlights the physiological basis of the diagnostic methods and comparatively evaluates their advantages and disadvantages in clinical practice.

### 4.1. Angiography-Based Techniques

Invasive coronary angiography (ICA) alone has limited sensitivity in detecting microvascular dysfunction.

**Thrombolysis in Myocardial Infarction (TIMI) Flow** and **Myocardial Blush Grade (MBG)** are complementary angiographic, qualitative techniques used to assess coronary and myocardial perfusion after revascularization. TIMI flow evaluates blood flow in the epicardial coronary arteries, but it is not sensitive enough to capture the adequacy of microvascular perfusion, even after full restoration of epicardial coronary flow to normal (TIMI flow grade 3) [45]. MBG assesses myocardial perfusion by grading the intensity and washout of contrast within the myocardium, with MBG: 0–1 indicating minimal or absent perfusion and MBG: 2–3 reflecting adequate perfusion. Notably, angiographic no-reflow, defined as reduced myocardial perfusion (TIMI flow ≤ 2 or MBG 0–1) despite successful epicardial coronary artery reopening, is closely related to CMVD and MVO [34,46].

**Corrected TIMI frame count (CTFC)** is another angiography-based technique to assess post-AMI CMVD measured by microvascular resistance during hyperemia [33]. However, all these angiography-based qualitative indices for CMVD diagnosis are significantly constrained by low accuracy and sensitivity, and high inter-observer and intra-observer variability [47].

**The index of microcirculatory resistance (IMR)** quantifies microvascular resistance using pressure and thermodilution techniques during hyperemia and is the most widely used method [48]. A guide catheter is inserted through the coronary orifice, and then the pressure and temperature are measured by an extending guidewire. After maximal hyperemia induced by adenosine or papaverine, the index is calculated using distal pressure and the mean transit time of 3 boluses of room temperature saline injected in the IRA (Infarct-Related Artery) [49]. IMR can be calculated using the formula IMR = Pa × Tmn × ([Pd − Pw]/[Pa − Pw]), where Pa = mean proximal coronary pressure, Tmn = mean hyperemic transit time, and Pd = mean distal coronary pressure. An IMR value above 25 is suggestive of CMVD, when the epicardial artery is patent. IMR is considered as the gold standard for invasive CMVD assessment, since it precisely quantifies microvascular dysfunction and may assist to stratify patients’ risk after AMI.

**Coronary flow reserve (CFR)** evaluates the ratio of maximal coronary blood flow during hyperemia to resting blood flow [46]. It can be measured invasively using either a pressure–temperature sensor-tipped guidewire or a Doppler catheter assessing blood flow velocity and resistance. Thermodilution-based CFR uses a pressure–temperature sensor guidewire, requiring at least three saline injections to measure the mean transit time (Tmn) at rest and during hyperemia. CFR is calculated as Tmn(rest)/Tmn (hyperemia), with a cut-off value of <2.0 indicating impaired vascular function at the distal territory of the left anterior descending artery (LAD). When the LAD is patent, microvascular dysfunction is inferred from low CFR (<2.0). Notably, the invasive measurement of CFR using either thermodilution or Doppler flow velocity is recommended by ESC and AHA/ACC guidelines (IIa) for patients with persistent symptoms and normal or moderately stenosed coronary arteries [50,51]. Carrick et al. (2016) failed to demonstrate the added value of CFR in STEMI patients where combined IMR and CFR calculation did not outperform IMR alone in risk stratification [52]. CFR’s utility is limited due to the increased resting flow in STEMI and its inability to isolate microvascular status from residual epicardial stenosis [53]. Transthoracic Doppler echocardiography, PET, or stress CMR may provide alternative, non-invasive measurements of CFR [54,55].

Using a pressure–temperature wire, the **resistive reserve ratio (RRR) can be calculated.** It assesses the functional reserve of coronary microvasculature by comparing resistance at baseline versus during hyperemia. It is calculated by CFR using the distal coronary pressure ratio between resting and hyperemic condition ([resting mean transit time/hyperemic mean transit time] × [resting distal coronary pressure/hyperemic distal coronary pressure]). In acute STEMI, IMR and RRR have been associated with MVO, IMH, infarct size, and clinical adverse outcomes [56]. A low RRR indicates impaired vasodilatory capacity of the microcirculation [57]. This method is particularly useful in identifying reduced microvascular responsiveness to PPCI in the early phase of AMI [58].

Similar to RRR, the **microvascular resistance reserve (MRR)** has recently been proposed for CMVD diagnosis. It is calculated as the ratio of basal microvascular resistance to hyperemic resistance (MRR = (CFR/FFR) × (Pa resting/Pa hyperemia) [59]. Despite its independency from epicardial resistance and autoregulation, the lack of established normal reference values, coupled with considerable variability between patients [59], has called into question the clinical application of the recently described MRR. A reduced MRR post-AMI reflects impaired functional reserve and the inability of the microcirculation to address increased demand [60]. More studies are needed to establish the cut-off values as well as the prognostic relevance of MMR in post-AMI patients.

As an alternative to pressure or temperature guidewire, the invasive Doppler-based methods have been proposed. They are widely used for CMVD by measuring coronary blood flow velocity and resistance [38]. These techniques provide real-time, dynamic assessments of microvascular function, but are limited by operator dependency and technical challenges. The key Doppler-based indices include **CFR, coronary flow capacity (CFC), and hyperemic microvascular resistance (HMR).** CFR evaluates the ratio of hyperemic to resting coronary flow velocity using Doppler method, reflecting microvascular reserve [61]. The advantage of this method is the assessment of micro- and micro-vascular coronary flow at the distal territory of LAD. However, it has significant disadvantages: (a) insights into both epicardial and microvascular function, (b) influenced by systemic hemodynamic changes, (c) lower prognostic value compared to hyperemic microvascular resistance in STEMI patients [40]. CFC combines maximum coronary flow measurements with CFR to assess the coronary circulation’s capacity. Therefore, it overcomes the limitations of the CFR but requires advanced imaging and technical expertise [41]. Notably, the assessment of a non-culprit vessel’s CFC in the setting of STEMI has been shown to improve risk stratification following reperfusion of the IRA [62]. HMR is measured using a guidewire equipped with a distal pressure sensor and Doppler crystal. It calculates the ratio of hyperemic mean distal pressure to Doppler-derived hyperemic average peak velocity (APV). HMR can be specific to the microvasculature and independent of systemic factors, but requires precise Doppler flow measurements and pharmacological hyperemia. HMR can identify CMVD in STEMI patients and predict adverse clinical outcomes [40]. Also, HMR measured after PCI predicts microvascular injury and impaired myocardial blood flow, with a threshold of 2.5 mmHg/cm/s demonstrating high sensitivity for high-risk patients. HMR provides immediate results after revascularization and may be a better predictor of adverse clinical outcomes compared to CFR. However, its measurement is technically challenging, with higher failure rates due to the need for high-quality Doppler signals, requiring further research to establish standardized cut-offs and enhance its prognostic utility.

Despite the high specificity of invasive techniques, their utility is often balanced against practical limitations such as cost, time, technical complexity, and patient risk. Invasive wire-based and sensor-based methods involve the induction of steady-state hyperemia, prolonging the procedure and further instrumentation of the IRA. Furthermore, they require operator expertise, limiting their widespread use. On the other hand, wire-based methods directly quantify the continuous absolute coronary flow (Q) and remain unaffected by systemic hemodynamic variations, ensuring high specificity [59]. Reduced absolute coronary flow indicates MVO or microvascular dysfunction, even when the epicardial arteries appear unobstructed in post-AMI patients [63]. Using Ohm’s law as the ratio of distal pressure to coronary flow, the continuous thermodilution technique may allow calculation of coronary microvascular resistance (Rμ) and identification of STEMI patients with significant microvascular dysfunction after PPCI [64]. The lack of specific therapy for CMVD means they are reserved for research settings, since their contribution to patient management is limited [65].

The emerging **angiography-derived computational flow dynamics** have been proposed for the assessment of coronary microcirculation. Without traditional wire-based techniques, these simple methods mainly use the **quantitative flow ratio (QFR)** [66]. **Angiography-derived IMR (IMRangio**) uses computational fluid dynamics applied to a three-dimensional reconstruction of the coronary artery derived from angiographic views during adenosine-induced steady-state hyperemia. The procedure is faster, though it depends heavily on high-quality angiographic imaging and computational accuracy [67]. Similarly, **non-hyperemic IMRangio (NH-IMRangio)** avoids the use of pharmacologic hyperemia, reducing patient discomfort with a good diagnostic accuracy in STEMI, but may not fully capture dynamic microvascular responses under stress [67]. Lastly, the computational protocol has a minimal procedural burden, but its clinical validation in certain scenarios remains too limited to ensure reliability and facilitate their widespread adoption [47,68]

### 4.2. Non-Invasive Methods

**Figure 2:**
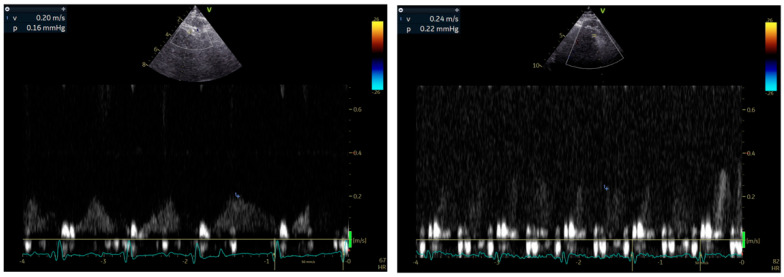
Representative example of CFR calculation using echocardiography.

**Stress echocardiography (SE)** remains a first-line technique for the diagnosis of significant CAD affecting epicardial coronary arteries. Wall motion abnormalities are usually not detected in patients with CMVD undergoing SE with dobutamine or exercise as stressors. Some operators have reported isolated hypokinesia of the apical segment of the interventricular septum, but this has not been established. Using the Doppler method and a modified apical view (between 2 and 3 chamber), **CFR can be measured during SE** as an index of either absolute coronary flow in the LAD or microvascular circulation patency [42] (Figure 2). CFR is calculated as the ratio of baseline to hyperemic coronary flow velocity, measured by pulsed-wave Doppler during diastole, with vasodilators like dipyridamole or adenosine inducing hyperemia. This is a widely used and easily accessible technique, which unfortunately cannot distinguish macrovascular from microvascular dysfunction. In patients with MINOCA only after the exclusion of significant stenosis in LAD, a low CFR (<2) indicates CMVD with impaired ability of the vasculature to dilate adequately and meet metabolic demands during hyperemia [57,69,70]. However, CFR is not directly associated with fractional flow reserve (FFR) due to its dependence on microvascular resistance [37]. This discrepancy explains why CFR and FFR are often discordant in approximately 30% of patients with intermediate coronary artery stenoses of the LAD [71]. Despite this, CFR complements FFR findings, offering additional insights into coronary microvascular physiology, especially when assessing the interplay between epicardial stenosis and microvascular function [37]. In daily clinical practice, the timing of applying SE after an AMI remains to be set.

**Myocardial perfusion in stress echocardiography** is a bedside technique that assesses microvascular perfusion using echo contrast agents. It can effectively detect MVO and “no-reflow” areas after AMI [43]. During myocardial contrast echocardiography (MCE), a high mechanical index impulse is applied to destruct all microbubbles of the contrast agent. In ischemic regions, a relatively delayed myocardial replenishment will cause a reduction in the contrast signal. Myocardial contrast signal intensity is directly proportional to blood volume. However, the adoption of this method is hindered by operator dependency, reproducibility, moderate spatial resolution, safety concerns, and reimbursement issues [72]. In a meta-analysis including 13 studies evaluating the diagnostic accuracy of quantitative MCE for detecting coronary artery disease (CAD), it was demonstrated that parameters reflecting CMVD, such microbubble velocity and myocardial blood flow, were significantly reduced in patients with CAD, highlighting the utility of MCE for assessing microvascular dysfunction in patients with CAD [73].

**CMR imaging** offers the capability to evaluate both global and artery-related CFR with the use of pharmacological vasodilators, like adenosine [74]. It is a safe, non-invasive method for evaluating myocardial perfusion and/or MVO. The latter is a subset of CMVD and a predictor of poor prognosis [75]. CMR enables direct visualization and quantification of MVO, reflecting myocardial damage due to CMVD, using first-pass perfusion (FPP) and LGE techniques [76]. Dobutamine stress CMR is usually performed using a 1.5 T system with cine imaging and myocardial tagging during rest and incremental dobutamine infusion, followed by a bolus injection of gadolinium-DTPA at peak-dose dobutamine to acquire FPP images [77]. Wall motion and perfusion images are then visually analyzed by an experienced cardiologist to identify myocardial ischemia based on perfusion deficits in at least two contiguous segments of consecutive short-axis slices [77]. CMR-derived indices of myocardial perfusion like microcirculatory perfusion index (MPI) and perfusion resistance index (MPRI) may be associated with invasive measurements of CMVD and hold prognostic significance [70]. Additionally, the administration of the gadolinium-based contrast agents enables both visual and semi-quantitative evaluation of myocardial perfusion reserve (MPR), identifying areas of impaired blood flow suggestive of CMVD. Novel techniques, like contrast-free T1 mapping, show potential in higher diagnostic accuracy for CMVD without reliance on gadolinium [78].

**Figure 3:**
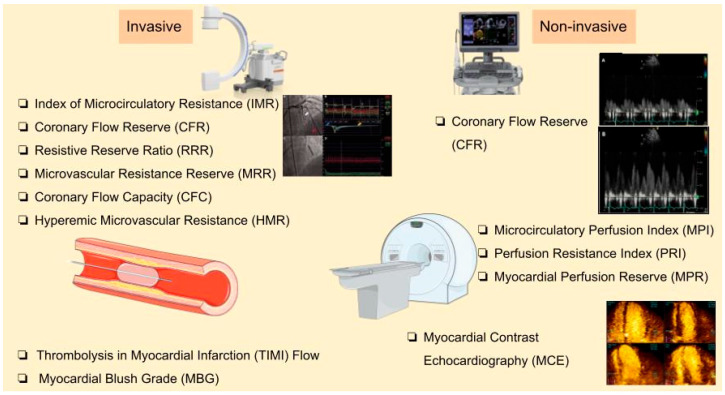
Invasive and non-invasive techniques for CMVD diagnosis.

**Single-photon emission computed tomography (SPECT)** and **cardiac PET/CT** are nuclear imaging techniques capable of detecting the no-reflow phenomenon in patients with MVO [79]. PET, with its ability to measure absolute myocardial blood flow and flow reserve, provides a non-invasive, quantitative assessment of myocardial perfusion by calculating CFR before and after administration of vasodilators, like adenosine [80,81]. However, PET scans remain underutilized, mainly due to limited availability of PET scanners and cyclotrons and higher cost [79]. A study by Mayala involving 28 patients demonstrated that PET/CT identified reduced CFR (<2.6) in 89% of patients with normal coronary angiography findings, confirming the high incidence of CMVD and highlighting its high sensitivity [81]. PET offers several advantages, including accurate quantification of myocardial blood flow and perfusion, and outperforms other modalities like SPECT by reducing false-positive results. It excels in quantifying coronary physiology and detecting CMVD, even in non-culprit coronary arteries, where reduced CFR has been shown to predict long-term adverse cardiovascular events [82]. Figure 3 depicts the available invasive and non-invasive techniques for CMVD.

### 4.3. Diagnostic Approach of Early Post-AMI CMVD

**Figure 4:**
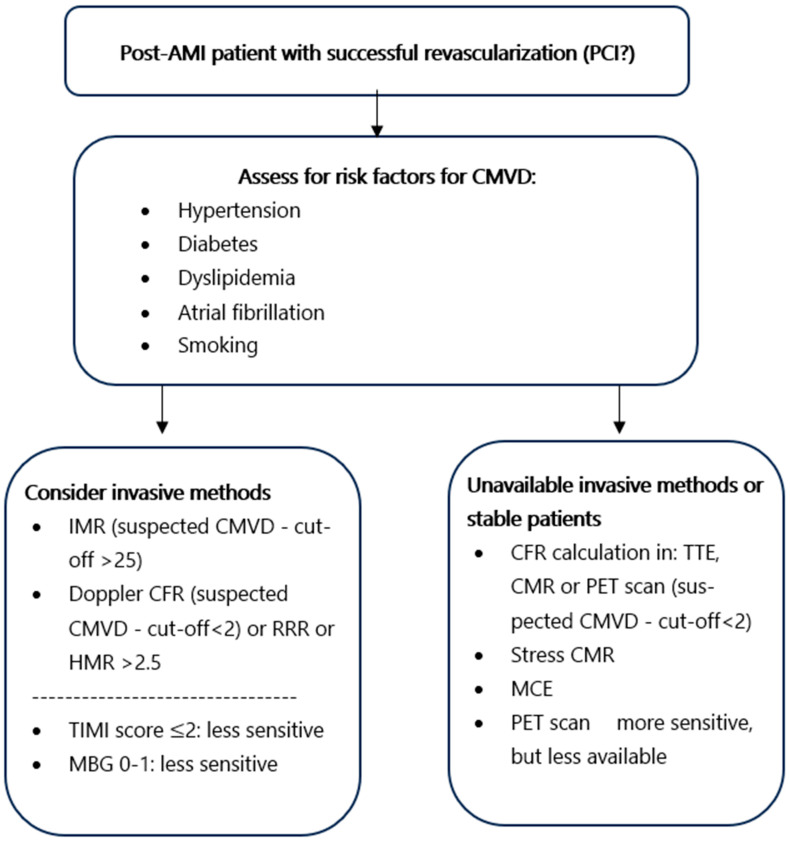
A diagnostic decision flowchart.

Each diagnostic modality for CMVD has distinct advantages and limitations and the diagnostic approach should be individualized. In patients with AMI undergoing PPCI, immediate invasive assessment of CMVD is appropriate. An IMR ≥ 25 suggests CMVD, and values > 40 in STEMI predict worse outcomes [83]. The angiographic measures of TIMI flow grade and MBG are immediately available, but with low sensitivity. If flow appears normal (TIMI 3 and MBG ≥ 2) [34], further testing may still be warranted due to the drawbacks of these modalities. CTFC may also reveal slow coronary flow, but it is less prognostically reliable. For a more precise assessment, microvascular resistance can be measured directly with pressure–wire-based indices [79]. HMR > 2.5 and a low RRR also indicate CMVD. These values help stratify risk and guide post-MI management. If invasive testing is not available or patients have been immediately stabilized, non-invasive modalities like PET, CMR, stress CMR, CFR from Doppler echocardiography, and MCE can be used. Transthoracic Doppler Echocardiography, while more accessible, has limitations related to operator dependency and image quality [84].These non-invasive techniques are typically carried out within a week post-MI. They provide detailed imaging of infarct size and MVO, strongly correlated with adverse outcomes [78]. Clinicians should carefully evaluate these factors in conjunction with patient-specific characteristics to determine the most suitable and safe diagnostic approach for CMVD (Figure 4).

## 5. Prognostic Value of CMVD

CMVD in post-AMI is linked to both poor prognosis and an impaired quality of life due to high morbidity, highlighting the clinical importance of accurate identification and diagnosis [85]. Over time, some of the diagnostic indices already mentioned in previous sections may not only provide valuable prognostic insights but also serve as a guide for therapeutic interventions to ameliorate the long-term outcomes of post-AMI patients [86,87].

### 5.1. Prognostic Value Based on Methods

**Myocardial contrast echocardiography (MCE):** Delayed or absent myocardial uptake at first pass of contrast agent indicates areas of no-reflow and CMVD. However, its widespread use is limited by the uncertain reproducibility, low sensitivity, and reimbursement challenges in some countries [43,88].

**CMR imaging:** Several indices of CMR have been analyzed for their potential prognostic value. Symons et al. demonstrated that the early detection of CMR-based CMVD is a strong independent predictor of MACE in patients after re-perfused STEMI [89]. As already mentioned, MVO extent ≥2.6% of the left ventricle was the strongest independent predictor of death and heart failure hospitalization up to six years after STEMI (HR: 3.12; 95% CI: 2.01–4.56; *p* < 0.0001) [85]. Although MVO may occupy only a small portion of the total infarct area (around 1–5% of the left ventricular mass in pooled studies), its extent correlates with the total infarct size and future events [90]. Notably, an early diagnosis of CMVD improves long-term risk stratification in patients with re-perfused STEMI [89]. The CMR-based assessment of both MVO and CFR relates to poor prognosis during follow-up after AMI [26,78]. A small study enrolling 30 patients after AMI showed that FPP combined with delayed enhancement in CMR (DE-CMR) effectively evaluated myocardial viability and MVO, which in turn were associated with ventricular remodeling and patient outcomes [91]. The same modality accurately identified IMH, associated with impaired ventricular function and infarct size at 1 month post-AMI, which apparently determined clinical outcomes during recovery [90].

**Cardiac PET studies:** Dysregulated post-infarction inflammation and remodeling is associated with adverse outcomes in post-AMI patients (HR: 3.76; 95% CI: 2.35–6.00; *p* < 0.001) [92]. A low CFR (<2) calculated via a PET scan has been associated with higher cardiac mortality risk [93]. PET-derived CFR provides a prediction of long-term cardiovascular outcomes. A previous clinical study of 49 STEMI patients undergoing PET scans to assess 18F-FDG uptake demonstrated that abnormal responses to sympathetic stimulation were linked to adverse functional outcomes like LVEF, LV end-systolic, and end-diastolic volume despite PPCI (HR: 2.89; 95% CI: 1.92–4.36; *p* < 0.0001) [44].

**Myocardial blush grade (MBG):** A recent meta-analysis showed a relationship of low MBG with larger infarct size, adverse ventricular remodeling, and worse outcomes in post-AMI patients [94]. MBG 0–1 has been associated with increased mortality at 16-month follow-up in AMI patients [11,95]. Kaya et al. demonstrated that MBG grade 3 serves as a significant marker for survival, superior to TIMI flow grades in the post-AMI follow-up period [96]. A meta-analysis of eight observational studies, encompassing 8044 patients with an overall low risk of bias, revealed that MBG grade: 0 or 1, indicative of poor or absent myocardial perfusion, carries a negative prognostic value for mortality (OR: 2.68; 95% CI [2.22–3.23]) and MACE (OR: 1.20; 95% CI [1.01–1.41]) in STEMI patients. Conversely, MBG grade 2 and 3, reflecting moderate and normal myocardial perfusion, respectively, were associated with improved survival outcomes (logHR: 0.47 (95% CI [0.43–0.52]) and log HR: 0.20 (95% CI [0.18–0.23], respectively) [94]. Despite the low sensitivity of MBG, it could be a prognostic marker for STEMI patients with CMVD.

**IMR:** Currently, IMR remains the gold standard invasive technique for CMVD diagnosis. Based on a growing body of data, it has been associated with MVO and adverse clinical events. IMR has also been used to predict the recovery of left ventricular function after elective PCI [35]. Interestingly, patients with STEMI and IMR >40 appeared with higher incidence of mortality and heart failure, and an 11.9-fold higher likelihood of having an infarct size greater than 25% of the myocardial mass at 6-month follow-up. This was further confirmed in another study of STEMI patients undergoing PPCI where the increased IMR due to MVO was independently associated with larger infarct size and poorer long-term clinical outcomes [HR: 4.2 (95% CI: 1.4–12.5); *p* = 0.009] [97]. In contrast, when IMR remained preserved, significant regression of infarct size was achieved over time. Moreover, high IMR demonstrated a modest but significantly negative correlation with LVEF at 3 months (*p* = 0.004) and at 1 year (*p* < 0.0001), which is of clinical relevance [98]. Maznyczka et al. recently demonstrated that in patients with STEMI presented within six hours of symptom onset, both RRR and IMR were associated with the extent of MVO, IMH, infarct size, and clinical outcomes [56].

The superiority of RRR (cut-off: <2.62) over CFR (cut-off: <2.5) has been proposed in patients with angina and non-obstructed coronary arteries, but further research is warranted in AMI [99]. On the other hand, the MRR offers a theoretical advantage by enabling more reliable inter-patient comparisons. However, it is a relatively novel index, and available data are limited, requiring validation. In a large cohort of 446 AMI patients undergoing PPCI, the low MRR cut-off for predicting the primary endpoint was set at (≤1.25). During a median follow-up of 3.1 years (Q1–Q3: 1.5–6.1 years), the composite outcome of all-cause mortality or heart failure hospitalization occurred in 27.3% vs. 5.9% of patients with low MRR value compared to those with higher (>1.25) (HR: 4.16; 95% CI: 2.31–7.50; *p* < 0.001) [100]. The independent prognostic value of MRR measured just after PPCI was also recently mentioned by Tsai TY et al. in 2024 [60].

**Hyperemic microvascular resistance (HMR):** In a cohort of 176 STEMI patients treated with successful PPCI, HMR was a strong predictor of both adverse clinical outcomes (death and heart failure hospitalization) and microvascular injury during a follow-up of 3.2 years (median value) [101]. This observation indicates that an increased HMR distal to a coronary stenosis reflects important pathophysiological alterations in the distal microvasculature in the setting of obstructive coronary artery disease. HMR may therefore be a useful tool to quantify the functional status of the myocardial microvasculature in clinical practice [102].

**IMRangio:** This novel, wire-free angiography-derived index has demonstrated good accuracy in predicting CMVD in the IRA of STEMI patients when compared with IMR and MVO [67]. In NSTEMI patients undergoing PCI, those with IMRangio > 25 exhibited a significantly higher incidence of MACEs (cardiac death, readmission for heart failure, myocardial reinfarction, and target vessel revascularization) compared to those with IMRangio ≤ 25 (32.52% vs. 9.37%; *p* < 0.001). Moreover, post-PCI IMRangio > 25 was identified as an independent predictor of MACEs (HR: 4.230; 95% CI: 3.151–5.679; *p* < 0.001). The addition of IMRangio to a predictive model using exclusively conventional risk factors improved the discriminatory ability [103].

**Coronary flow reserve (CFR)**: Despite its limited capability to assess coronary microcirculation in the presence of residual epicardial stenosis, an abnormal CFR has still been linked to a significantly higher incidence of MACEs in patients with acute coronary syndromes (HR: 3.76; 95% CI: 2.35–6.00) [104]. Two recent meta-analyses, comprising 19 and 11 studies, respectively, demonstrated that low CFR in patients with myocardial ischemia but with non-obstructive CAD—evaluated through either non-invasive techniques (SE, positron emission tomography-PET), or invasive methods—was significantly associated with an increased risk of death and MACE [105,106]. Moreover, CFR measured with either non-invasive or invasive methods was significantly associated with final infarct size and reduced LVEF in STEMI patients undergoing angiographically successful PPCI [107]. The early measurement of CFR by TTE can assess the degree of successful reperfusion in AMI and can predict LV functional recovery, myocardial viability, and the final infarct size [55].

**ECG findings:** Lack of rapid ST-segment resolution (STR) in STEMI, despite successful PPCI, does not necessarily indicate failure to recanalize the artery. Instead, it often signifies an inability to restore adequate myocardial perfusion, typically due to CMVD [108,109].

### 5.2. Other Prognostic Considerations and Pharmaceutical Implications

The timing of microvascular dysfunction assessment post-AMI significantly impacts the pathophysiological interpretation and prognostic implications. Acute-phase microvascular dysfunction predominantly reflects immediate ischemia-reperfusion injury, endothelial dysfunction, and inflammation, which are crucial early determinants of infarct size and short-term clinical outcomes. In contrast, late-phase dysfunction often represents persistent endothelial impairment, chronic inflammation, and structural remodeling leading to progressive ventricular remodeling and heart failure risk [110]. Practically, immediate invasive indices like IMR and RRR allow early stratification of patients at risk of adverse outcomes immediately after PPCI. However, these invasive procedures, while highly specific, are technically demanding and carry potential risks. Non-invasive methods such as CMR and PET offer safer, albeit less accessible, alternatives providing significant prognostic insights into long-term outcomes and microvascular integrity [39]. Implementing these diagnostic assessments routinely in clinical practice could aid clinicians in tailored management of patients post-AMI, improving individualized patient care. However, challenges like cost, accessibility, operator expertise, and patient risk profile need careful consideration for optimal integration into clinical workflows (Table 2).

Effective management of CMVD requires targeting underlying mechanisms such as endothelial dysfunction, oxidative stress, inflammation, and microvascular remodeling [111]. Pharmacologic therapies should target diverse pathophysiological pathways. The 2023 guidelines recommend beta-blockers as first-line therapy for symptom relief in CMVD patients, while calcium channel blockers are considered for those who do not respond adequately to beta-blockers or have contraindications [112]. ACE inhibitors or ARBs are suggested for patients with hypertension or other compelling indications, aiming to improve endothelial function. Statins are advocated for lipid management and their pleiotropic effects on the microvasculature, including reducing oxidative stress and inflammation. Nevertheless, more robust evidence is required to advocate for these pharmaceutical therapies in the early phase of myocardial infarction.

Novel therapies such as SGLT2 inhibitors exhibit cardioprotective effects beyond glycemic control, improving endothelial function and reducing microvascular inflammation [113]. Endothelin receptor antagonists (e.g., bosentan) and anti-inflammatory agents (e.g., colchicine) are also being explored for their potential to address endothelial dysfunction and persistent inflammation [114,115]. The use of intracoronary adenosine or nicorandil during PCI could reduce no-reflow and enhance microvascular perfusion. Thrombus aspiration and distal embolic protection devices minimize distal embolization [116]. The coronary sinus reducer, a novel device, shows promise in improving microvascular perfusion in refractory angina by modulating venous outflow [49,117]. Gene and stem cell therapies aim to regenerate damaged microvasculature [118], while novel anti-inflammatory agents (e.g., canakinumab) target specific inflammatory pathways. Metabolic modulators (e.g., trimetazidine, ranolazine) enhance myocardial energy metabolism, potentially reducing microvascular ischemia [119,120]. The current pharmaceutical data are limited and have been derived from patients with stable CAD. Future studies should investigate the clinical efficacy and prognostic benefits of these emerging therapies in CMVD after AMI.

## 6. Conclusions and Future Perspectives

Since the presence of CMVD is associated with an increased risk of mortality and MACE even after a successful revascularization in individuals with AMI, several invasive and non-invasive imaging modalities have been proposed to enhance the accuracy of CMVD diagnosis in the early phase of AMI. Invasive calculation of CFR through thermodilution or Doppler flow velocity are already recommended by ESC and AHA/ACC guidelines (IIa) in stable patients. In particular, eligible patients are those with chronic CAD and episodes of chest pain, but normal or moderately stenosed coronary arteries [51]. Currently, their implementation as a routine practice for AMI patients is limited in research papers. The measurement of IMR during catheterization could be a possible way to promote CMVD investigation in all AMI patients. Unambiguously, this technique provides high specificity and modest sensitivity for CMVD diagnosis, but, on the other hand, it is time-consuming, requiring hyperemia provocation during an acute phase of AMI.

The echocardiography-based techniques mainly used to measure the CFR remain an alternative to invasive methods. The presence of residual epicardial stenosis can interfere with the precision of the measurements. During the early post-infarction period, the identification of CMVD with CMR could be useful for individuals with high probability, but negative results for obstructed coronary arteries. It remains an independent predictor of long-term morbidity. Lastly, PET scans could be reserved for patients with high probability of CMVD, but PET’s availability and cost issues limit this modality as a last-resort option. Furthermore, the timing of performing any of these tests is a significant issue, since, shortly after the AMI (up to 6 weeks), a maximum-stress test is contraindicated.

It is essential that all available indices should be validated through large prospective cohort studies and head-to-head comparisons. There are numerous medications that seem to be effective regarding the management of CMVD [32]. Also, medications administered during PCI such as adenosine, calcium channel blockers, aspirin, unfractionated heparin, potent P2Y12 inhibitors or nicorandil glycoprotein IIb/IIIa inhibitors, or low-dose thrombolytics could be effective in minimizing the risk of CMVD development [121]. It is evident that the improvement of survival and quality of life for AMI patients is largely influenced by the combination of various medications targeting multiple pathophysiological pathways.

## Figures and Tables

**Table 1:** A comparative evaluation of methods for CMVD diagnosis early after acute myocardial infarction.

Diagnostic Method	Pathophysiology	Clinical Insights and Evaluation
Invasive		
**TIMI Flow Grade (≤2) [33]**	Reduced blood flow in angiography cine loops	**Clinical outcomes**: Association with in-hospital mortality and adverse events. **Pros**: Simple calculation, cost-effective, performed during every PCI. **Cons**: Qualitative, poor reproducibility, does not directly assess microvascular function.
**Myocardial Blush Grade (≤1) [34]**	Reduced myocardial perfusion grading the intensity and washout of contrast within the myocardium during angiography	**Clinical outcomes**: Association with infarct size and adverse remodeling. **Pros**: Insights into myocardial perfusion. **Cons**: Subjective interpretation, limited sensitivity and specificity.
**Corrected TIMI Frame Count (CTFC) [33]**	Delayed contrast transit in angiography measured by frame count indicates impaired perfusion	**Clinical outcomes**: Linked to worse outcomes compared to those without CMVD. **Cons**: Time-consuming, technical confounders.
**Index of Microcirculatory Resistance (IMR > 25) [35]**	Elevated microvascular resistance assessed via thermodilution during hyperemia	**Clinical outcomes**: Association with MACEs. **Pros**: Quantitative gold standard of invasive methods, reproducible, specific to microvascular function. **Cons**: Hyperemia induction, specialized equipment, and expertise.
**Angiography-derived IMR (IMRangio) (>25) [36]**	Computational fluid dynamics model based on angiographic images simulates IMR	**Clinical outcomes**: Strong correlation with invasive IMR and MVO. Independently predicts MACEs post-PCI in STEMI and NSTEMI. **Pros**: Wire-free, faster, no hyperemia needed (in NH-IMRangio), lower procedural risk. **Cons**: Depends on image quality and validated software, limited real-world data, less validated than invasive IMR.
**Coronary Flow Reserve (CFR < 2, invasive) [37]** CFR (Doppler < 2.1) [38]	Impaired capacity of coronary circulation to augment flow during hyperemia Reduced coronary flow velocity reserve using Doppler guidewire	**Clinical outcomes**: Association with MACEs. **Pros**: Evaluates both epicardial and microvascular function. **Cons**: Cannot distinguish between epicardial and microvascular dysfunction, influenced by hemodynamic variables.
**Resistive Reserve Ratio (RRR ≤ 2.62) [39]**	Reduced functional vasodilatory reserve of microvasculature during stress-induced hyperemia	**Clinical outcomes**: Association with adverse events. **Pros**: Insights into microvascular functional reserve. **Cons**: Invasive measurements and hyperemia induction.
**Hyperemic Microvascular Resistance (HMR ≥ 2.5 mmHg/cm/s) [40]**	Increased resistance to flow during hyperemia at distal coronary microvasculature	**Clinical outcomes**: Association with adverse events. **Pros**: Specific assessment of microvascular resistance. **Cons**: Technically challenging, hyperemia induction and Doppler expertise required.
**Coronary Flow Capacity (CFC < 2.8) [41]**	Combined impairment in both CFR and absolute flow capacity	**Clinical outcomes**: Association with adverse events. **Pros**: Comprehensive assessment of whole coronary flow. **Cons**: Requires advanced imaging techniques and expertise.
**Non-invasive**		
**Transthoracic Doppler Echocardiography (TTE) CFR (<2) [42]**	Reduced coronary flow velocity in LAD measured by Doppler echocardiography during hyperemia	**Clinical outcomes**: Association with MACEs. **Pros**: Non-invasive, easily accessible. **Cons**: Operator-dependent, limited in patients with suboptimal acoustic windows.
**Myocardial Contrast Echocardiography (MCE) [43]**	Uses microbubble contrast agents to visualize myocardial perfusion, assessing microvascular integrity and perfusion defects	**Clinical outcomes**: Association with adverse events. **Pros**: Bedside applicability, real-time imaging. **Cons**: Limited availability, expertise required.
**Cardiac Magnetic Resonance (CMR) with MVO ≥ 2.6% of LV mass [26]**	Detects microvascular obstruction and perfusion abnormalities using gadolinium contrast	Limited data available
PET CFR (<2.0–2.6) [44]	Decreased hyperemic myocardial blood flow on PET indicating microvascular dysfunction	**Clinical outcomes**: Strong predictor of MACEs. **Pros**: Quantitative gold standard of non-invasive methods. **Cons**: High cost, limited availability.

**Abbreviations:** CFC: Coronary Flow Capacity; CFR: Coronary Flow Reserve; CMR: Cardiac Magnetic Resonance; CTFC: Corrected TIMI Frame Count; HMR: Hyperemic Microvascular Resistance; IMR: Index of Microcirculatory Resistance; IMRangio: Angiography-Derived Index of Microcirculatory Resistance; LAD: Left Anterior Descending Artery; LV: Left Ventricle; MACE: Major Adverse Cardiovascular Events; MBG: Myocardial Blush Grade; MCE: Myocardial Contrast Echocardiography; MPI: Microcirculatory Perfusion Index; MRPI: Myocardial Perfusion Reserve Index; MVO: Microvascular Obstruction; PCI: Percutaneous Coronary Intervention; PET: Positron Emission Tomography; RRR: Resistive Reserve Ratio; STEMI: ST-Segment Elevation Myocardial Infarction; TIMI: Thrombolysis in Myocardial Infarction; TTE: Transthoracic Echocardiography.Adverse events: hospitalization for heart failure and/or acute coronary syndromes MACEs: any single parameter or any combination of the following: mortality, hospitalization for heart failure, and/or acute coronary syndromes.

**Table 2:** Assessing the prognostic value of both invasive and non-invasive indices during the early phase of myocardial infarction.

Index	Studied Population	Timing	Methodology	Clinical Outcomes in Patients with CMVD
**TIMI Flow (≤2) [33]**	STEMI patients	Immediate post-reperfusion	Angiographic qualitative assessment of coronary flow	↑ In-hospital mortality
**MBG (≤1) [94]**	AMI patients	Immediate post-PCI	Qualitative myocardial perfusion assessment via angiography	↑ Infarct area, adverse remodeling, ↑ hospitalization for heart failure
**IMR (>25, or >40) [68]**	AMI patients post-PCI	Immediate post-reperfusion	Thermodilution-based coronary microvascular resistance measurement	Predictive of MVO, ↑ infarct size, ↑ MACE, ↑ mortality
**CFR invasive (<2) [37]**	AMI patients	Immediate post-PCI	Pressure–temperature guidewire measurement of coronary flow velocity	↑ In-hospital mortality
**CFR (<2.1, Doppler) [42]**	AMI patients	Post-reperfusion	Doppler-based coronary flow measurement	↑ Cardiac mortality
**RRR (≤1.5, alternatives: 1.7, 2.62) [39]**	STEMI patients with PPCI	Immediate post-procedure	Functional reserve assessment of coronary microvasculature	↑ infarct size, ↑ MACE, ↑ mortality
**CFC (<2.8) [62]**	AMI patients	Post-reperfusion	Combines coronary flow measurements with CFR	↑ risk stratification, ↑ MACE
**HMR (≥3) [102]**	STEMI patients	Post-primary PCI	Measurement combining hyperemic distal pressure and Doppler velocity	↑ hospitalization for heart failure, ↑ MACE, ↑mortality
**MCE [43]**	AMI patients	Post-reperfusion	Echocardiographic myocardial contrast imaging	↑ Infarct area
**CMR: MVO (≥2.6), MPI, MRPI [90]**	AMI patients	Post-reperfusion	Magnetic resonance imaging to visualize myocardial perfusion	↑ Infarct area, ↑ hospitalization for heart failure
**PET-derived CFR (<2.6, alternative: 2.0) [93]**	AMI patients	Post-infarction	Positron emission tomography for myocardial flow quantification	↑ Long-term cardiovascular events and mortality

↑, increased.

## Data Availability

Data are contained within the article.

## Abbreviations

The following abbreviations are used in this manuscript:

18F-FDG	Fluorodeoxyglucose F-18
AHA/ACC	American Heart Association/American College of Cardiology
ACS	Acute Coronary Syndrome
AMI	Acute Myocardial Infarction
ATP	Adenosine Triphosphate
CAD	Coronary Artery Disease
CDKN2B-AS1	Cyclin-Dependent Kinase Inhibitor 2B Antisense RNA 1
CFC	Coronary Flow Capacity
CFR	Coronary Flow Reserve
CFVR	Coronary Flow Velocity Reserve
CI	Confidence Interval
CMVD	Coronary Microvascular Disease
CMR	Cardiac Magnetic Resonance
CTFC	Corrected TIMI Frame Count
DAMPs	Damage-Associated Molecular Patterns
DE-CMR	Delayed Enhancement Cardiac Magnetic Resonance
ESC	European Society of Cardiology
FFR	Fractional Flow Reserve
FPP	First-Pass Perfusion
HF	Heart Failure
HMR	Hyperemic Microvascular Resistance
HR	Hazard Ratio
IL-6	Interleukin-6
IMH	Intramyocardial Hemorrhage
IMR	Index of Microcirculatory Resistance
IMRangio	Angiography-Derived Index of Microcirculatory Resistance
INOCA	Ischemia with Non-Obstructive Coronary Arteries
IRA	Infarct-Related Artery
LAD	Left Anterior Descending Artery
LGE	Late Gadolinium Enhancement
LV	Left Ventricular
LVEF	Left Ventricular Ejection Fraction
MACE	Major Adverse Cardiovascular Events
MBG	Myocardial Blush Grade
MCE	Myocardial Contrast Echocardiography
MINOCA	Myocardial Infarction with Non-Obstructed Coronary Arteries
MPI	Microcirculatory Perfusion Index
MPRI	Myocardial Perfusion Reserve Index
MRR	Microvascular Resistance Reserve
MVO	Microvascular Obstruction
MYH15	Myosin Heavy Chain 15
NH-IMRangio	Non-Hyperemic Angiography-Derived Index of Microcirculatory Resistance
NO	Nitric Oxide
NT5E	5′-Nucleotidase Ecto
OR	Odds Ratio
PCI	Percutaneous Coronary Intervention
PET	Positron Emission Tomography
PPCI	Primary Percutaneous Coronary Intervention
QFR	Quantitative Flow Ratio
ROS	Reactive Oxygen Species
RRR	Resistive Reserve Ratio
SE	Stress Echocardiography
SPECT	Single-Photon Emission Computed Tomography
STEMI	ST-segment Elevation Myocardial Infarction
STR	ST-segment Resolution
TTE	Transthoracic Echocardiography
TIMI	Thrombolysis in Myocardial Infarction
TNF-α	Tumor Necrosis Factor-alpha
VEGFA	Vascular Endothelial Growth Factor A
